# Effect of supercritical carbon dioxide fluid extract from *Chrysanthemum indicum* Linné on bleomycin-induced pulmonary fibrosis

**DOI:** 10.1186/s12906-021-03409-9

**Published:** 2021-09-25

**Authors:** Juan Nie, Yanlu Liu, Chaoyue Sun, Jingna Zheng, Baoyi Chen, Jianyi Zhuo, Ziren Su, Xiaoping Lai, Jiannan Chen, Jibiao Zheng, Yucui Li

**Affiliations:** 1 School of Pharmaceutical Sciences, Guangzhou University of Chinese Medicine, 510006 Guangzhou, China; 2 2nd Clinical Hospital of Guangzhou University of Chinese Medicine, Guangzhou, 510120 China; 3 Guangdong Provincial Key Laboratory of New Drug Development and Research of Chinese Medicine, Guangzhou University of Chinese Medicine, Guangzhou, 510006 China; 4 Department of Pharmacy, Central People’s Hospital of Zhanjiang, Zhanjiang, 524000 China

**Keywords:** *Chrysanthemum indicum* Linné, Supercritical carbon dioxide extraction, Idiopathic pulmonary fibrosis, Wnt/β-catenin signalling pathway

## Abstract

**Background:**

As a prevalent type of cryptogenic fibrotic disease with high mortality, idiopathic pulmonary fibrosis (IPF) still lacks effective therapeutic drugs. The compounds extracted from buds and flowers of *Chrysanthemum indicum* Linné with supercritical-carbon dioxide fluid (CI_SCFE_) has been confirmed to have antioxidant, anti-inflammatory, and lung-protective effects. This paper aimed to clarify whether CI_SCFE_ could treat IPF induced by bleomycin (BLM) and elucidate the related mechanisms.

**Methods:**

Rats (Sprague-Dawley, male) were separated into the following groups: normal, model, pirfenidone (50 mg/kg), CI_SCFE_-L, −M, and -H (240, 360, and 480 mg/kg/d, i.g., respectively, for 4 weeks). Rats were given BLM (5 mg/kg) via intratracheal installation to establish the IPF model. A549 and MRC-5 cells were stimulated by Wnt-1 to establish a cell model and then treated with CI_SCFE_. Haematoxylin-eosin (H&E) and Masson staining were employed to observe lesions in the lung tissues. Quantitative real-time polymerase chain reaction (qRT-PCR) and Western blot (WB) were performed to observe changes in genes and proteins connected with the Wnt/β-catenin pathway.

**Results:**

CI_SCFE_ inhibited the proliferation of MRC-5 cells (IC_50_: 2.723 ± 0.488 μg/mL) and A549 cells (IC_50_: 2.235 ± 0.229 μg/mL). In rats, A549 cells, and MRC-5 cells, BLM and Wnt-1 obviously induced the protein expression of α-smooth muscle actin (α-SMA), vimentin, type I collagen (collagen-I), and Nu-β-catenin. The mRNA levels of matrix metalloproteinase-3 (MMP-3) and − 9 (MMP-9), two enzymes that degrade and reshape the extracellular matrix (ECM) were also increased while those of tissue inhibitor of metalloproteinase 1 (TIMP-1) were decreased. However, CI_SCFE_ reversed the effects of BLM and Wnt-1 on the expression pattern of these proteins and genes.

**Conclusion:**

These findings showed that CI_SCFE_ could inhibit IPF development by activating the Wnt/β-catenin pathway and may serve as a treatment for IPF after further investigation.

**Supplementary Information:**

The online version contains supplementary material available at 10.1186/s12906-021-03409-9.

## Background

Idiopathic pulmonary fibrosis (IPF), a prevalent fibrotic disease with high mortality, is a complex pathology of the lung with unknown aetiology [1, 2]. Previous studies have reported that inflammation occurs early following lung injury [3]. Excess inflammation and abnormal repair result in epithelial-mesenchymal transition (EMT) [4] and abnormal growth of lung interstitial cells, resulting in changes in the collagens deposed into the extracellular matrix (ECM) [5]. However, as IPF is a pulmonary disease characterized by progressive interstitial fibrosis, parenchymal inflammation and accumulation of ECM protein [6], the detailed cellular and molecular mechanisms of its development remain unknown. Currently, the main treatment strategies of IPF include glucocorticoids, antifibrinolytic agents, and antioxidants. The most commonly used drug is pirfenidone (PFD). However, PFD also has inevitable side effects [7, 8]. Thus, new potential targets and agents to ameliorate IPF urgently need to be identified and developed.

The Wnt signalling pathway participates in self-renewal of stem cells, metabolic disease, bone disease, and cancer [9]. Based on the different types of downstream effectors, there are two Wnt signalling pathways: the canonical pathway (Wnt/β-catenin) and the non-canonical pathway [10]. Emerging evidence has shown that the canonical pathway plays an active role in IPF development [11]. β-Catenin, as a necessary element of the canonical pathway, participates in the physiological process of EMT, which is a major process involved in fibrotic tissue repair after injury, tumour progression, and embryonic development [12]. The Wnt protein is critical in the canonical pathway. When lung tissue is damaged, Wnt protein expression can be stimulated, and subsequent downstream signal inhibits the phosphorylation of β-catenin and slows its degradation. Therefore, β-catenin accumulates in the cytoplasm and then translocates to the nucleus, where it regulates the expression of interrelated genes, including matrix metalloproteinases (MMPs) [13]. Moreover, it is known that matrix metalloproteinase-3 (MMP-3) and matrix metalloproteinase-9 (MMP-9) can degrade various ECM closely interrelated with the occurrence of IPF [14]. Therefore, the Wnt/β-catenin signalling pathway may affect the occurrence of IPF by affecting ECM deposition.

*Chrysanthemum indicum* Linné, a medicinal and edible plant, is mostly used in pharmaceutical products and in health products, beverages, and food additives. Clinically, it has been used to treat coronary heart disease, hypertension, and respiratory diseases such as pneumonia and bronchitis [15]. Many experimental data have confirmed that the extract of *Chrysanthemum indicum* Linné has antitumour [16], antioxidant [17], antimicrobial [18], anti-inflammatory, and immunomodulatory effects [19, 20]. The supercritical carbon dioxide extraction is widely used to extract chemical constituents from *Chrysanthemum indicum* Linné, not only because the supercritical carbon dioxide extraction is efficient and environmentally friendly, but also because it can obtain more biologically active compounds and ensure the structural integrity of volatile compounds. Specifically, the extract from buds and flowers of *Chrysanthemum indicum* Linné in supercritical carbon dioxide fluid (CI_SCFE_) has been widely used in cosmetics, functional foods, and toiletries [21]. Moreover, our previous study showed that it could enhance the antitumour effect and reduce pulmonary damage of bleomycin (BLM) [22]. In addition, CI_SCFE_ has been demonstrated to protect against UV-induced skin injury and lipopolysaccharide (LPS)-induced lung injury [23, 24] We previously observed that CI_SCFE_ can significantly inhibit inflammatory cytokines induced by LPS and produced by alveolar epithelial cells, thereby alleviating LPS-mediated lung injury [24]. When CI_SCFE_ and BLM were administered together to treat tumours, we found that BLM combined with CI_SCFE_ could remarkably attenuate collagen deposition and inflammatory damage in lung tissues. Based on the above findings, we hypothesized that CI_SCFE_ has a lung protective effect. However, whether CI_SCFE_ could be used to treat IPF remains unknown.

In this study, to determine whether CI_SCFE_ could relieve or treat pulmonary fibrosis, we used a BLM-induced IPF model and Wnt-1-induced cell model to verify this hypothesis.

## Methods

### Antibodies and reagents

DMEM, RPMI 1640 medium, foetal bovine serum (FBS) and phosphate-buffered solution (PBS) was provided by Gibco (Grand Island, NY, USA). Hydroxyproline (HYP) assay kits were obtained from Shanghai Kejian Biology Science and Technology Co., Ltd. (Shanghai, China). Penicillin-streptomycin was provided by HyClone (Logan, UT, USA). Poly-clonal antibodies targeting GAPDH (AF7021), Histone H3 (AF0863), α-smooth muscle actin (α-SMA, AF1032), vimentin (AF7013), β-catenin (AF6266), and type I collagen (collagen-I, AF7001) as well as HRP-labelled Goat Anti-Rabbit lgG antibodies (E030120–01) were obtained from Affinity Biosciences (OH, USA).

### Drugs

We dissolved BLM (Zhejiang Hai Zheng Pharmaceuticals, China. purity> 99%) in 0.9% normal saline. CI_SCFE_ (Lot. 20,121,104) was manufactured by the Mathematical Engineering Academy of Chinese Medicine at the Guangzhou University of Chinese Medicine. We analysed the CI_SCFE_ composition using high-performance liquid chromatography with a photodiode array detector (HPLC-PAD) and gas chromatography-mass spectrometry (GC-MS) [24]. GC-MS detected thirty unique compounds, and HPLC-PAD identified five compounds (Supplementary Materials 1). CI_SCFE_ was suspended in 0.9% normal saline containing 3% Tween 80 as a cosolvent. Pirfenidone was provided by Dalian Meilun Biological Technology Co., Ltd. (lot number: A0730A; Dalian, China). Normal saline (0.9%) was used to dissolve pirfenidone.

### Procedure for supercritical-carbon dioxide fluid extraction

*Chrysanthemum indicum* Linné was purchased from Guangzhou Qingping medicinal materials market (Guangzhou, China), authenticated based on its microscopic and macroscopic characteristics. *Chrysanthemum indicum* Linné was placed in the extraction kettle (5 L-SFE, Guangzhou Institute of Light Industry), and then reflux extraction for 3 h under the condition of 25 MPa, 45 °C and the flow rate of CO_2_ was 30 kg/h.

### Cells

The Cell Bank of the Chinese Academy of Science provided the MRC-5 cells (human lung fibroblasts cell line) and A549 cells (adenocarcinoma of human alveolar epithelial cell line). Cells were cultured in DMEM/RPMI 1640 medium, which included 1% penicillin-streptomycin and 10% FBS, at 37 °C and 5% CO_2_.

### Cytotoxicity assay

A549 cells (1 × 10^4^ cells/well) and MRC-5 cells (0.75 × 10^4^ cells/well) were seeded in 96-well plates for MTS detection. Then, the medium was replaced with medium containing CI_SCFE_ (20, 40, 60, 80, 100, 200, 400, 800, or 1000 ng/mL). MTS (20 μL) was employed after cells were cultured for 24/48 h. Four hours later, cell viabilities were measured at 492 nm using a multimode plate reader. The IC50 of CI_SCFE_ in the two cell lines was calculated using GraphPad Prism software (version 6).

The effects of different doses of Wnt-1 (5, 10, 15, 20, 30, 40, 60, and 100 ng/mL) on A549 and MRC-5 cells were also studied using MTS. Cells were seeded into 96-well plates determine cell viability. Serum-free medium was added when the cells adhered. Then, the cells were stimulated with 20 ng/mL Wnt-1 for 24 h after serum starvation for 6 h. Then, the cells were treated with medium containing 400 ng/mL CI_SCFE_. Cells were assessed after 24 h of treatment.

### Experimental animal procedures and pulmonary fibrosis model establishment

The Experimental Animal Centre of Guangzhou University of Chinese Medicine (Certificate number 44005800005378, Guangzhou, China) provided male adult Sprague-Dawley (SD) rats. The procedures were conducted under the guidance of the Animal Care and Welfare Committee of Guangzhou University of Chinese Medicine. Rats were placed in an SPF environment (temperature 24–26 °C, humidity 70–75%). Rats were provided free access to food and water. The animal experiments were conducted according to the guidelines established by the National Institutes of Health (NIH) Guide for the Care and Use of Laboratory Animals.

Sixty rats were randomly assigned to the following six groups (*n* = 10; group by random number table method): normal, model, PFD, CI_SCFE_-L, −M, and -H. Except for rats in the control group, all the rats were given BLM (5 mg/kg) via intratracheal installation to establish a pulmonary fibrosis model. Three days after BLM injection, intragastric administration was started. Normal saline was administered to rats in the normal and model groups. PFD (50 mg/kg) was given to rats in the PFD group, and rats in the CI_SCFE_-L, −M, and -H groups were treated with CI_SCFE_ (240, 360, and 480 mg/kg, respectively). All drugs were intragastrically administered once per day. During the experiment, none of the rats died.

After 28 days, all rats were weighed and administered 1% pentobarbital sodium (40 mg/kg) via intraperitoneal injection. Then, the rats were sacrificed by bleeding from the abdominal aorta, and the lung tissues were rapidly collected and washed with ice-cold normal saline. The lung tissues were weighed, and the left lung tissues (0.1 g) of all rats were fixed with 4% paraformaldehyde. The remaining lung tissue was stored at − 80 °C.

### Relative lung weight and body weight changes

The relative lung weight was calculated as follows: lung coefficient = lung weight (g)/body weight (kg) × 100%.

### Histological analysis

After fixation for 24 h, the fixed lung tissue was dehydrated with different concentrations of alcohol, and different xylenes were used for transparency. Then, lung tissues were embedded in paraffin. After the paraffin was cooled, 5 μm sections were cut to observe inflammatory infiltration and collagen deposition with haematoxylin-eosin (H&E) and Masson’s trichrome staining, respectively. Then, the stained sections were observed under a microscope. The grades of pathologic changes that indicated lung injury were evaluated referencing others’ reports [25, 26]. Lung injury includes oedema, congestion, inflammatory cell infiltration, and interstitial inflammation, each of which was graded from 0 to 4. The scores of each category per individuals were added to the final score of lung injury.

### HYP analysis

Lung tissues (0.1 g) were homogenized in HYP Assay Kit hydrolysate using a tissue homogenizer. After halting the hydrolysate reaction according to the manufacturer’s instructions, the absorbance of the supernatant was measured at 550 nm on a spectrophotometer.

### Cell transfection and treatment

MRC-5 (1 × 10^5^ cells/mL) and A549 (1.25 × 10^5^ cells/mL) cells were seeded into 6-well plates. Then, the cells were stimulated with Wnt-1 (20 ng/mL) when they reached 70–80% confluence. After incubation for 24 h, cells were treated with medium containing CI_SCFE_ (400 ng/mL). RNA and protein were extracted after 24 h.

The small interfering RNA (siRNA) targeting β-catenin and the negative control were provided by RiboBio (Guangzhou, China). Lipofectamine 2000 (Invitrogen) was adopted to transfect β-catenin siRNA (40 nM) or negative control siRNA into the cells according to the manufacturer’s instructions. Then, the cells were treated as described above.

### Quantitative real-time polymerase chain reaction (qRT-PCR)

We extracted total RNA from two kinds of cells and lung tissues with TRIzol reagent (Invitrogen) according to the manufacturer’s instructions. Ultra-micro spectrophotometer (Thermo Fisher Scientific, USA) was used to measure the purity and concentration of extracted RNA. When the OD260/OD280 value was in the range of 1.8 ~ 2.1, the RNA purity is considered to meet the experimental requirements. A reverse transcription kit (Vazyme Biotech Co., Ltd., China) was applied to reverse transcribe total RNA. The cDNA of two cells and lung tissues were stored at − 20 °C. Then, qRT-PCR amplification was accomplished using a ChamQ™ Universal SYBR qPCR Master Mix Kit (Vazyme Biotech Co., Ltd., China) under the following cycling conditions: 95 °C for 30 s followed by 40 cycles of 95 °C for 10 s, 60 °C for 30 s, and 72 °C for 60 s. The respective primers are listed in Table 1. GAPDH served as the reference gene. This study employed the 2^-∆∆Ct^ method to calculate gene expression based on the following formula: Fold change = 2^-∆∆Ct^, ∆∆Ct = (Ct_Sample_ - Ct_GAPDH_) - (Ct_Control_ - Ct_GAPDH_).

**Table 1:** Primers sequences employed for quantitative PCR

Species	Gene	ID	Forward (5′ - 3′)	Reverse (3′ - 5′)
Human	GAPDH	2597	GGCACCGTCAAGGCTGAGAAC	CAGGTACGGTAGTGACGGTGG
	MMP-3	4314	CGGTTCCGCCTGTCTCAAGATG	GAGAAGGAAGTCCGCACCTACG
	MMP-9	4318	GAATGGCATCCGGCACCTCTATG	GGAAGGAATAGCGGCTGTTCACC
	TIMP-1	7076	TGGCTTCTGGCATCCTGTTGTTG	CCTCTCACAGACGCCTATGAAGGT
Rat	GAPDH	24,383	GTCCATGCCATCACTGCCACTC	GTTCTTCCACCACTTCGTCCGC
	MMP-3	171,045	GGGAAGCTGGACTCGAACACT	GGATAAGGACCAACGACGAGT
	MMP-9	81,687	CAAACCCTGCGT ATTTCCATT	CGTTGAGCCGTCCTCTCTACA
	TIMP-1	116,510	TTTGCATCTCTGGCCTCTG	CGCAATACTCTAGTTCTAC

### Western blotting

Total proteins were extracted from two kinds of cells and lung tissues with radio-immunoprecipitation assay (RIPA) buffer, and the proteins from the nuclear and cytoplasmic fractions of two cells and lung tissues were obtained with a Nuclear and Cytoplasmic Protein Extraction Kit (Keygen Biotech, Jiangsu, China). The protein concentrations of all the samples were estimated using the Bicinchoninic Acid Protein Kit (Best Bio, Shanghai, China). Fifty micrograms of two cells and lung tissues proteins were resolved using SDS-PAGE on 12% or 8% gels and were transferred onto PVDF membranes (Millipore, Billerica, USA). The membranes were blocked with 5% non-fat milk for 1 h. The membranes were incubated with anti-GAPDH (1:1000), anti-H3 (1:1500), anti-α-SMA (1:500), anti-vimentin (1:500), anti-β-catenin (1:500) and anti-collagen-I (1:500) antibodies overnight at 4 °C. Then, the membranes were treated with secondary antibodies (1:2000) for 2 h. Protein expression of two cells and lung tissues were measured by a chemiluminescence system (Tanon). The band densities of GAPDH and H3 were used as a reference.

### Statistical analysis

SPSS 23.0 software was used to analyse the results, and the data are expressed as the means ± SD. Duncan’s test and one-way ANOVA were used to analyse the statistical significance among different groups. *P* < 0.05 was considered statistically significant.

## Results

### Effects of Wnt-1 and CI_SCFE_ on MRC-5 and A549 cells

**Fig. 1:**
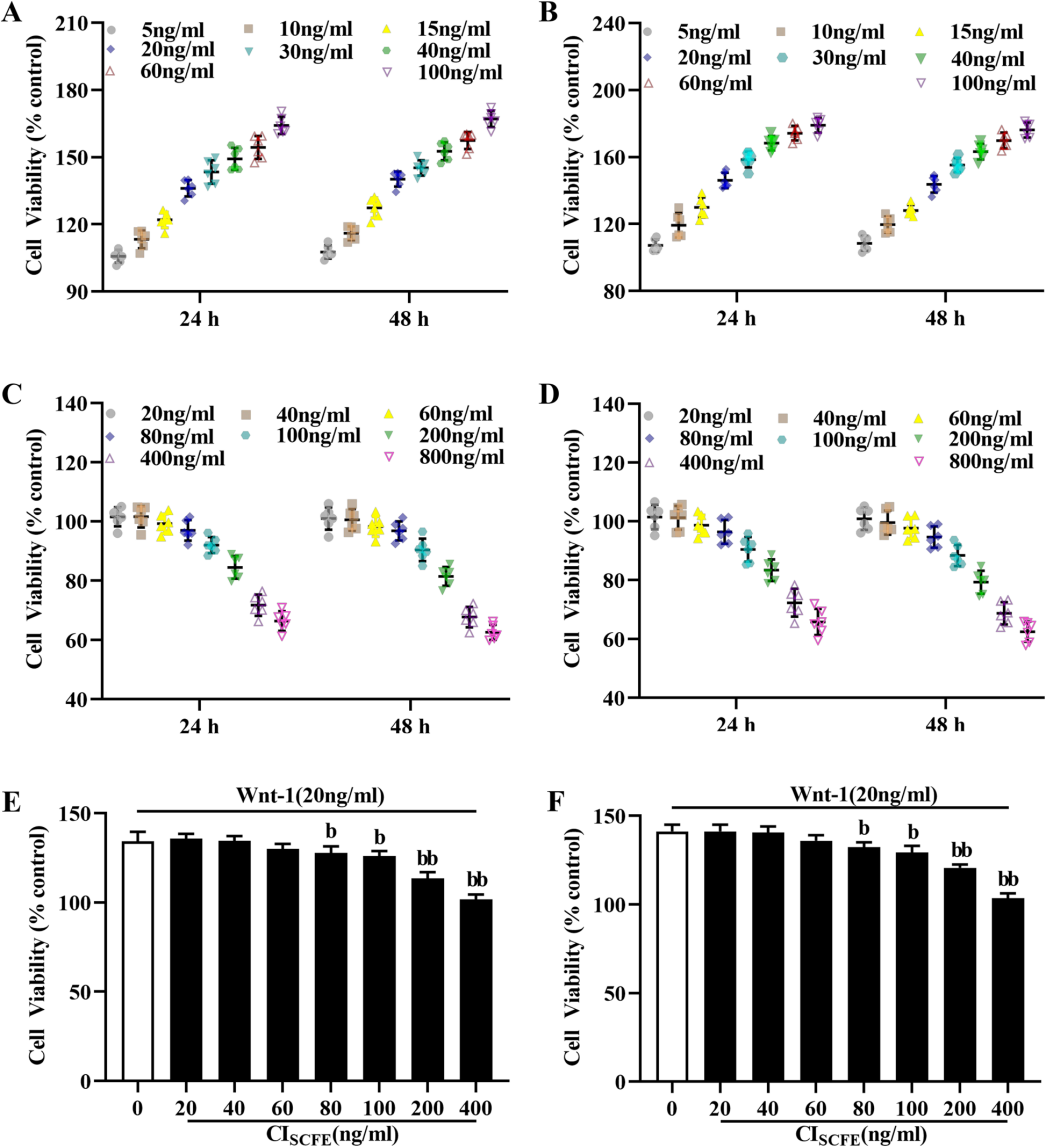
Effects of Wnt-1 and CI_SCFE_ on A549 and MRC-5 cells. Effects of treatment with Wnt-1 (5, 10, 15, 20, 30, 40, 60, or 100 ng/mL) on A549 (**A**) and MRC-5 cells (**B**). Effects of treatment with CI_SCFE_ (20, 40, 60, 80, 100, 200, 400, 800, and 1000 ng/mL) on A549 (**C**) and MRC-5 cells (**D**). The viability of A549 cells (**E**) and MRC-5 cells (**F**) was measured after stimulation with Wnt-1 (20 ng/mL) for 24 h followed by administration of CI_SCFE_ (20, 40, 60, 80, 100, 200, and 400 ng/mL). Cell viability is presented as the mean ± SD. (*n* = 6). ^a^, *P* < 0.05; ^aa^, *P* < 0.01 vs the control group. ^b^, *P* < 0.05 ^bb^, *P* < 0.01 and ^bbb^, *P* < 0.001 vs the Wnt-1 group

Figure 1 shows that the growth rates of A549 (1A) and MRC-5 cells (1B) were obviously faster after cells were treated with Wnt-1 (5, 10, 15, 20, 30, 40, 60, and 100 ng/mL) for 24 h or 48 h. However, CI_SCFE_ significantly inhibited the proliferation of A549 cells (IC_50_: 2.723 ± 0.488 μg/mL) (1C) and MRC-5 cells (IC_50_: 2.236 ± 0.230 μg/mL) (1D).

Figure 1E, F shows that compared with the Wnt-1 alone group, the CI_SCFE_ (80, 100, 200, 400 ng/mL) groups showed significantly reduced cell viability (*P* < 0.05). The data illustrated that CI_SCFE_ can significantly suppress Wnt-1-mediated proliferation of A549 cells and MRC-5 cells. Based on these results, Wnt-1 (20 ng/mL) and CI_SCFE_ (400 ng/mL) were utilized for subsequent experiments.

### Effect of CI_SCFE_ on relative lung weight and level of HYP in BLM-treated rats

**Fig. 2:**
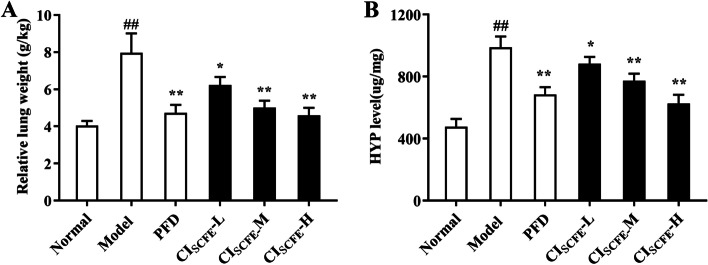
Effect of CI_SCFE_ on the relative lung weight and HYP level of BLM-treated rats. **A** Relative lung weight of BLM-treated rats. **B** Effect of CI_SCFE_ on HYP levels in BLM-treated rat lung tissues. The results are shown as the means ± SD. (*n* = 8). ^##^, *P* < 0.01 vs the normal group; ^*^, *P* < 0.05 and ^**^, *P* < 0.01 vs the model group

We analysed the effect of CI_SCFE_ on the experimental rat lung coefficient. BLM upregulated the lung coefficient (*P* < 0.05) (Fig. 2A). However, after CI_SCFE_ treatment, the lung coefficient value of rats significantly decreased.

HYP is the principal constituent of collagen. The degree of IPF can be reflected by the content of HYP. Figure 2B shows that CI_SCFE_-M and -H treatment can reduce the HYP level.

### Effect of CI_SCFE_ on IPF in BLM-treated rats

**Fig. 3:**
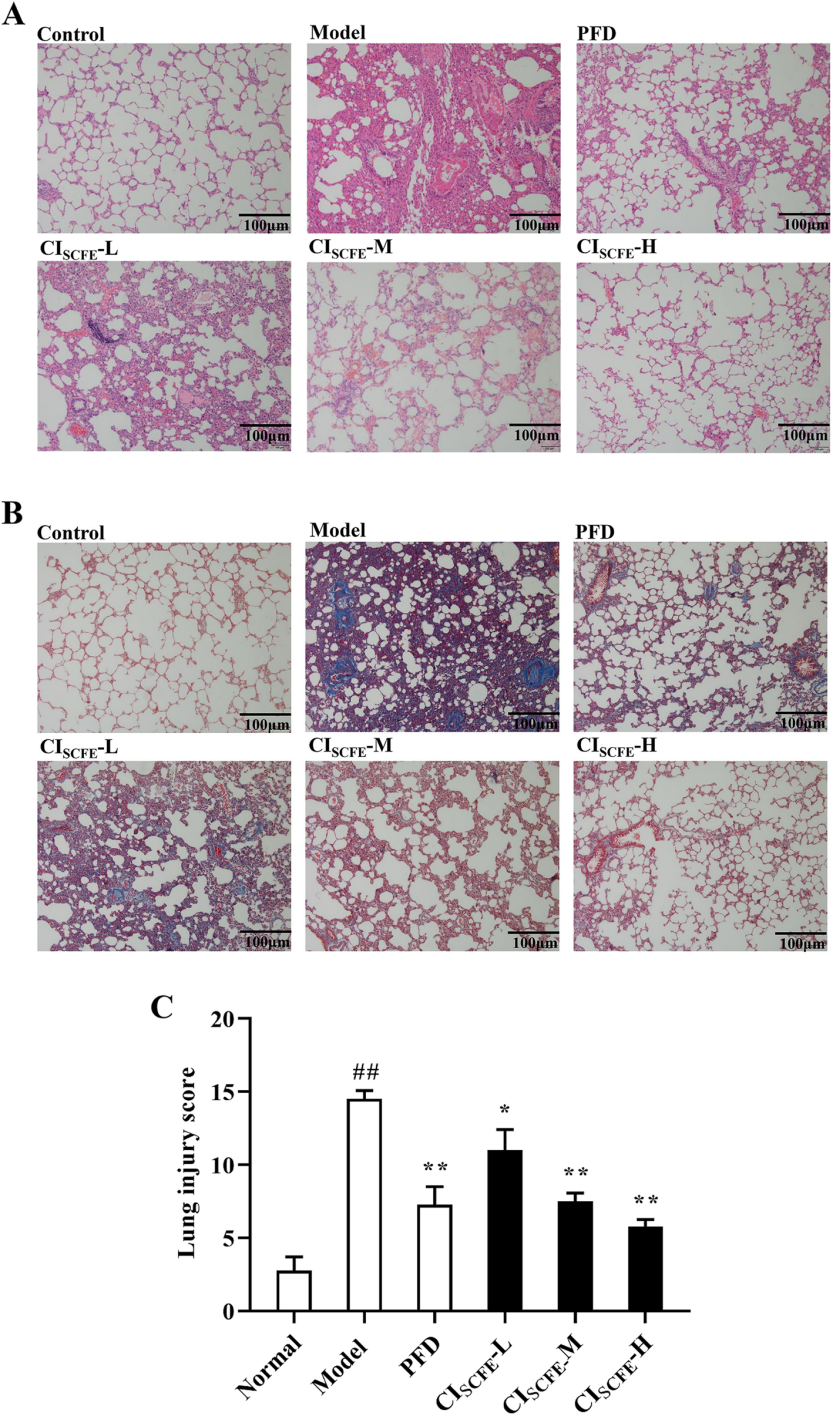
Effect of CI_SCFE_ attenuated BLM-induced IPF. **A** Observation of inflammation (× 200). **B** Observation of collagen deposition (× 200). **C** Lung injury score. Scale bar indicates 100 μm. The results are shown as the means ± SD. (*n* = 4). ^##^, *P* < 0.01 vs the normal group; ^*^, *P* < 0.05 and ^**^, *P* < 0.01 vs the model group

The model group showed significant inflammation, collagen deposition and severely damaged lung tissue structure (Fig. 3A, B). The CI_SCFE_-M and -H groups showed that the above situation was relieved after treatment compared with the model group. Figure 3C shows that BLM can obviously induce lung injury in rats. However, CI_SCFE_-M and CI_SCFE_-H alleviated the lung injury caused by BLM in rats (*P* < 0.05). These results indicated that CI_SCFE_ can alleviate IPF caused by BLM to a certain extent.

### Effect of CI_SCFE_ on the expressions of α-SMA, Vimentin, collagen-I, nu-β-catenin in A549 cells, MRC-5 cells and BLM-treated rats

**Fig. 4:**
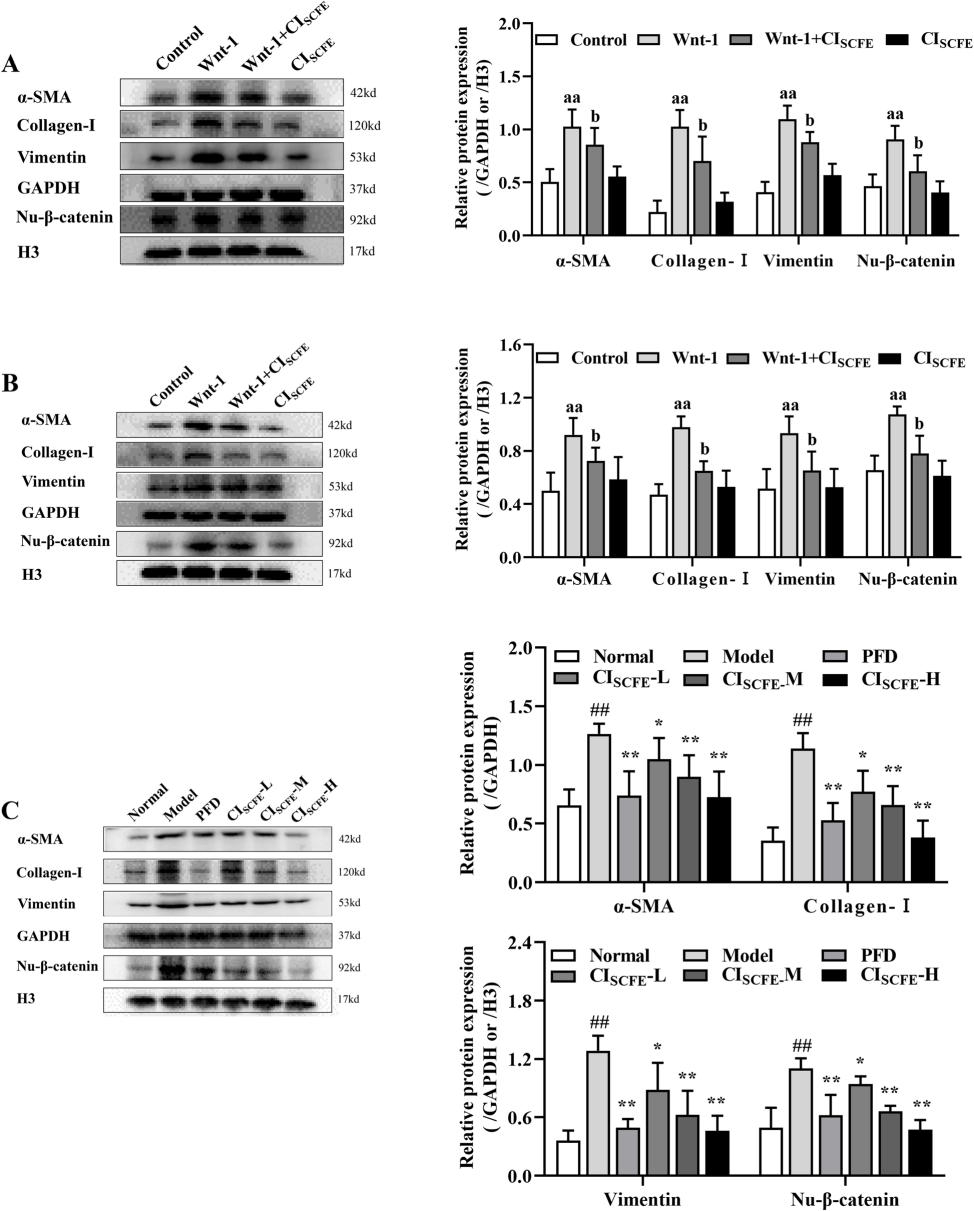
Effect of CI_SCFE_ on α-SMA, Vimentin, Collagen-I, Nu-β-catenin expression in A549, MRC-5 cells and BLM-treated rats. **A** Effect of CI_SCFE_ on the expression of α-SMA, Vimentin, Collagen-I, Nu-β-catenin in A549 cells. **B** Effect of CI_SCFE_ on the expression of α-SMA, Vimentin, Collagen-I, and Nu-β-catenin in MRC-5 cells. **C** Effect of CI_SCFE_ on the expression of α-SMA, Vimentin, Collagen-I, and Nu-β-catenin in BLM-treated rat lung tissues. The relative expression of Nu-β-catenin is shown as the ratio relative to H3 expression. The results are presented as the mean ± SD. (*n* = 3). To fit into the manuscript properly, the gel was reasonably trimmed. ^aa^, *P* < 0.01 vs the control group; ^b^, *P* < 0.05, ^bb^, *P* < 0.01 vs the Wnt-1 group. ^##^, *P* < 0.01 vs the normal group; ^*^, *P* < 0.05 and ^**^, *P* < 0.01 vs the model group

In this study, BLM obviously increased α-SMA, vimentin, collagen-I and Nu-β-catenin protein expression in A549 cells (Fig. 4A) and MRC-5 cells **(**Fig. 4B) (*P* < 0.05). The expression of these proteins was downregulated in A549 and MRC-5 cells treated with CI_SCFE_. In addition, Fig. 4C shows that the expression of the above proteins in BLM-treated rat lung tissues was decreased in the CI_SCFE_-M and -H groups (*P* < 0.05). Thus, CI_SCFE_ relieved the abnormal deposition of ECM proteins in BLM-treated rats.

### Effect of CI_SCFE_ on MMP-3, MMP-9 and TIMP-1 mRNA levels in A549 cells, MRC-5 cells and BLM-treated rats

**Fig. 5:**
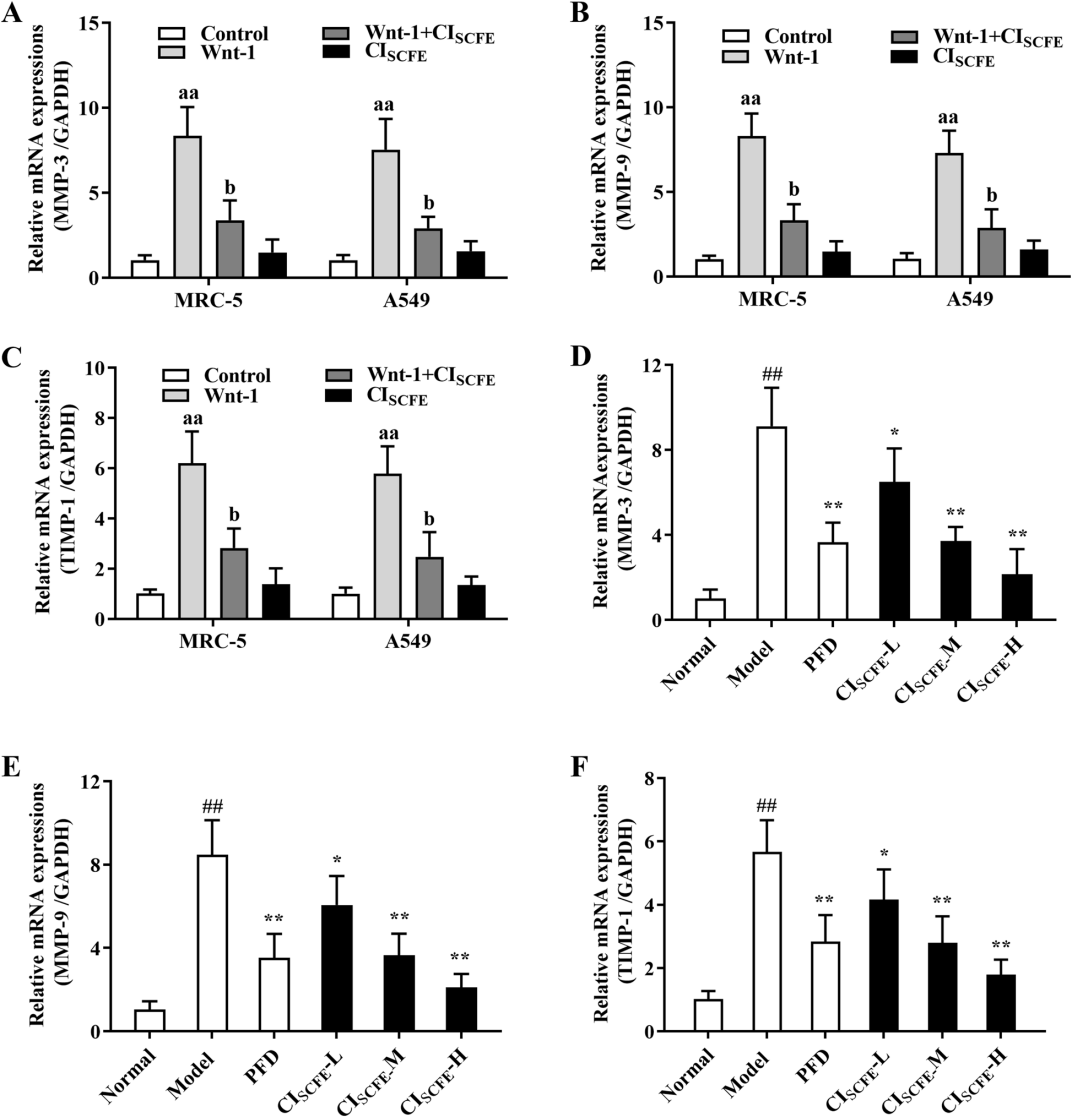
Effect of CI_SCFE_ on MMP-3, MMP-9 and TIMP-1 mRNA levels in A549 cells, MRC-5 cells and BLM-treated rats. Effect of CI_SCFE_ MMP-3 (**A**), MMP-9 (**B**) and TIMP-1 (**C**) mRNA levels in A549 and MRC-5 cells was observed. The influence of the CI_SCFE_ on MMP-3 (**D**), MMP-9 (**E**) and TIMP-1 (**F**) mRNA levels in BLM-treated rats was noted. The results are presented as the means ± SD. (*n* = 6). ^aa^, *P* < 0.01 vs the control group; ^b^, *P* < 0.05 and ^bb^, *P* < 0.01 vs the Wnt-1 group. ^##^, *P* < 0.01 vs the normal group; ^*^, *P* < 0.05 and ^**^, *P* < 0.01 vs the model group

A549 cells and MRC-5 cells treated with CI_SCFE_ showed downregulation of MMP-3 (Fig. 5A), MMP-9 (Fig. 5B), and TIMP-1(Fig. 5C) mRNA levels (*P* < 0.05), when compared with Wnt-1 group. After treatment with CI_SCFE_-M or CI_SCFE_-H, MMP-3 (Fig. 5D) and MMP-9 (Fig. 5E), and TIMP-1(Fig. 5F) mRNA levels were decreased (*P* < 0.05) in BLM-treated rats, compared with model group. The data showed that IPF induced by BLM can be relieved by CI_SCFE_ by affecting the gene expression associated with abnormal deposition of collagen in the ECM.

### Effect of CI_SCFE_ on the Wnt/β-catenin Signalling pathway in A549 and MRC-5 cells following β-catenin siRNA knockdown

**Fig. 6:**
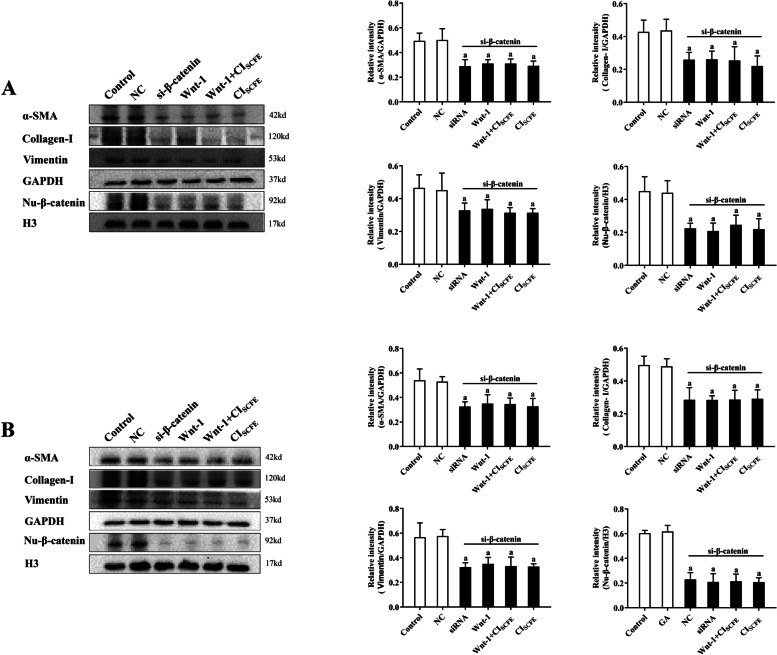
Effect of CI_SCFE_ on the Wnt/β-catenin signalling pathway in A549 and MRC-5 cells following siRNA-mediated β-catenin knockdown. **A** Effect of CI_SCFE_ on the Wnt/β-catenin signalling pathway in A549 cells. **B** Effect of CI_SCFE_ on the Wnt/β-catenin signalling pathway in MRC-5 cells. The results are presented as the means ± SD. (n = 3). To fit into the manuscript properly, the gel was reasonably trimmed. ^a^, *P* < 0.05 vs control group

Figure 6 shows that α-SMA, vimentin, collagen-I, and Nu-β-catenin protein expression were unchanged following β-catenin siRNA knockdown in A549 cells (Fig. 6A) and MRC-5 cells (Fig. 6B) when cells were treated with Wnt-1 (20 ng/mL) for 24 h. After treatment with CI_SCFE_, the expression of the above proteins was also unchanged in both cell lines when β-catenin was knocked down compared with the wnt-1 group. The data illustrated that CI_SCFE_ adjusted the Wnt/β-catenin signalling pathway to alleviate the process of IPF.

## Discussion

IPF is a pulmonary interstitial disease with a high mortality rate. Currently, both the morbidity and mortality of IPF has increased year after year. Even the mortality rate is higher than that of most tumours. However, owing to the poorly understood potential pathogenesis of IPF, there is still a lack of drugs that can completely cure IPF. Our previous study illustrated that CI_SCFE_ can improve the antitumour effect of BLM while simultaneously attenuating its toxicity effects and CI_SCFE_ could inhibit acute lung injury induced by LPS [22, 24, 27]. This current study clarified that CI_SCFE_ can inhibit IPF development by affecting the balance of the Wnt/β-catenin pathway.

IPF is caused by a variety of factors. The idea that abnormal ECM deposition is a cardinal feature of pulmonary fibrosis has been generally accepted. The ECM, which comprises collagens, proteoglycans, elastin, and other molecules, is involved in proper lung function. Myofibroblasts have been confirmed to contribute to ECM accumulation in vivo and in vitro [28]. Studies have found that both epithelial cells and fibroblasts in the lungs can transform into myofibroblasts through EMT, which is the process by which epithelial cells transform into mesenchymal cells. According to molecular and functional characterization, EMT is divided into three categories: type I EMT related to embryogenesis, type II EMT linked to fibrosis and wound healing and type III EMT involved in cancer metastasis [29]. It is generally accepted that type II EMT has a substantial effect on the development of IPF [30, 31]. In addition, studies have shown that the occurrence of EMT is accompanied by an increase in the expression of a mesenchymal phenotype marker (vimentin) and abnormal deposition of ECM proteins, such as α-SMA and collagen-I [32, 33]. MRC-5 cells, a cell line that retains the biochemical characteristics of lung fibroblasts, are commonly utilized as lung fibroblast cells in pulmonary fibrosis research [34, 35]. Although the A549 cell line is a human-derived lung adenocarcinoma cell line, it is widely used to establish an EMT model by stimulating the cells with Wnt-1 or other cytokines [36, 37] because its morphology and basic cell functions are similar to those of human alveolar type II epithelial cells. Therefore, to investigate whether CI_SCFE_ could affect the transformation of epithelial cells and fibroblasts to myofibroblasts in vitro, we used A549 cells and MRC-5 cells to establish a cell model. MRC-5 cells were treated with Wnt-1 to establish a cell transdifferentiation model, and A549 cells were stimulated by Wnt-1 to establish an EMT model. Our experimental results indicated that CI_SCFE_ could significantly decrease vimentin, α-SMA and collagen-I expression in A549 cells, MRC-5 cells, and lung tissues of BLM-induced IPF rats. The data illustrated that BLM-induced IPF could be inhibited by CI_SCFE_ via suppression of EMT and abnormal ECM deposition.

MMPs and tissue inhibitors of metalloproteinases (TIMPs) have been reported to participate in the formation and degradation of the ECM. MMPs are multidomain enzymes that have outstanding roles during the cleavage of constituents of the ECM. Twenty-four MMP genes have been identified in humans, and 8 MMPs have been implicated in IPF development. Not only did in vitro studies indicate that there was high expression of MMP-3 in the alveolar epithelial cells and lungs of IPF patients, but in vivo studies have also confirmed this phenomenon. Researchers observed high expression of MMP-9 in the lung tissues of IPF patients. Additionally, TIMPs are endogenous inhibitors that control the catalytic activity of MMPs. Usually, under normal physiological conditions, the ratio of MMPs to TIMPs is approximately 1:1, but under pathological conditions, this balance may be destroyed, leading to excessive deposition of ECM components [38]. Therefore, an imbalance of MMPs/TIMPs is associated with IPF. Studies have shown that not only MMP-3 levels but also MMP-9 levels were increased in IPF lungs [39]. TIMP-1 levels were also upregulated in IPF lungs but to a lesser extent than those of MMP-3 [40]. Y. Wang et al. [41] found that artesunate could inhibit the occurrence of IPF by decreasing MMP-3, MMP-9 and TIMP-1 levels. Hai-Tao Zhang et al. [42] confirmed that the balance of MMP-9/TIMP-1 expression in IPF patients was disrupted, and this ratio was obviously higher than that in normal patients. After glucocorticoid intervention, the MMP-9/TIMP-1 ratio in IPF patients was restored closer to homeostatic levels, and the MMP-9 and TIMP-1 levels were reduced. The experimental data indicated that CI_SCFE_ could significantly decrease MMP-3 and MMP-9 mRNA levels in the lung tissues of BLM-induced IPF rats and in A549 and MRC-5 cells. At the same time, the level of TIMP-1 gene expression could also be downregulated by CI_SCFE_. The MMP/TIMP ratio was also decreased after CI_SCFE_ treatment. These data illustrated that CI_SCFE_ could inhibit the progression of BLM-induced IPF by regulating the imbalance of MMPs/TIMPs, which facilitates the elimination of abnormal ECM deposition.

Even though we know that EMT and the ECM are closely related to IPF development, we still have a poor understanding of the clear mechanism by which EMT and the ECM influences IPF. Recently, compelling evidence has revealed that Wnt/β-catenin reactivation is connected with EMT and the ECM [43, 44]. The Wnt/β-catenin pathway participates in adult stem cell maintenance, embryonic development and homeostasis [45, 46]. However, its abnormal activation leads to the progression of carcinomas of the liver, colon, lung and other organs [47–49]. Recently, some studies have shown that its abnormal activation is also involved in fibrotic diseases, such as IPF and renal fibrosis [13, 50, 51]. Therefore, Wnt/β-catenin signalling is considered a promising new target in the treatment of fibrotic disease [52]. In addition, the Wnt/β-catenin pathway was shown to be involved in the process of ECM deposition and EMT induction. Therefore, we speculated that CI_SCFE_ could inhibit the progression of BLM-induced IPF by downregulating the balance of the Wnt/β-catenin pathway. Studies have confirmed that β-catenin is a critical element of the Wnt/β-catenin pathway [53]. When external factors stimulate the tissue, β-catenin translocates to and accumulates in the nucleus, thereby activating the entire Wnt/β-catenin pathway. Van et al. [54] confirmed that β-catenin accumulated in the cell nucleus of lung tissues from IPF patients. Liang [55] also confirmed that during myofibroblast proliferation, β-catenin levels would increase in the nuclei of lung tissue cells. Our results showed that in the cell nuclei of lung tissues, β-catenin expression can be improved by BLM. However, CI_SCFE_ could inhibit this phenomenon. Based on the above results, we hypothesize that CI_SCFE_ affects IPF by regulating the balance of Wnt/β-catenin pathway activity.

To confirm the hypothesis that CI_SCFE_ could inhibit the progression of BLM-induced IPF by downregulating Wnt/β-catenin pathway activation, we used MRC-5 cells and A549 cells to investigate whether CI_SCFE_ could affect BLM-induced IPF after knockdown of the β-catenin gene. The β-catenin gene is a key gene in the Wnt/β-catenin pathway which will only be activated when β-catenin transferred into the cell nucleus. Therefore, we knockdown single gene β-catenin to block the activation of Wnt/β-catenin pathway. Human β-catenin siRNA was transfected into MRC-5 and A549 cells. The study results illustrated that CI_SCFE_ could suppress vimentin, α-SMA, β-catenin and collagen-I expression in Wnt-1-treated MRC-5 and A549 cells. By contrast, in MRC-5 and A549 cells transfected with β-catenin siRNA, CI_SCFE_ did not affect vimentin, α-SMA, β-catenin or collagen-I expression levels. The results revealed that CI_SCFE_ could inhibit the progression of BLM-induced IPF by influencing the balance of the Wnt/β-catenin pathway.

Our study still has some limitations. The pathogenesis of IPF is quite complicated. In this study, we investigated whether CI_SCFE_ inhibited IPF by downregulating Wnt/β-catenin pathway activity. However, we have not studied whether the attenuating effect of CI_SCFE_ is related to other signalling pathways. In addition, we have not studied the specific components in CI_SCFE_ that elicited these changes. Therefore, further research regarding these issues is necessary, and we will continue to study the issues related to the attenuating effect of CI_SCFE_ on IPF.

## Conclusions

CI_SCFE_ could alleviate IPF induced by BLM. The studies demonstrated that CI_SCFE_ could alleviate EMT by adjusting the balance of the Wnt/β-catenin pathway and ultimately attenuate BLM-induced IPF. These results illustrated that after further research, CI_SCFE_ could become a potential drug for IPF.

## Supplementary Information

**Additional file 1.**

**Additional file 2.**

**Additional file 3.**

## Data Availability

The data set used and analyzed in the study can be obtained from the corresponding author on reasonable request.

## Abbreviations

BLM: Bleomycin
CI_SCFE_: The extract extracted from buds and flowers of *Chrysanthemum indicum* Linné with supercritical-carbon dioxide fluid
Collagen- I: Type I collagen
EMT: Epithelial-mesenchymal transition
ECM: Extracellular matrix
HYP: Hydroxyproline
IPF: Idiopathic pulmonary fibrosis
LPS: Lipopolysaccharide
MMP-3: Matrix metalloproteinase-3
MMP-9: Matrix metalloproteinase-9
PFD: Pirfenidone
RT-PCR: Reverse Transcription-Polymerase Chain Reaction
SD: Sprague-Dawley
TIMP-1: Tissue inhibitor of metalloproteinase 1
WB: Western blot
α-SMA: α-smooth muscle actin

## References

[1] Katzenstein ALA, Myers JL (1998). Idiopathic pulmonary fibrosis - clinical relevance of pathologic classification. Am J Respir Crit Care Med.

[2] Barkauskas CE, Noble PW (2014). Cellular mechanisms of tissue fibrosis. 7. New insights into the cellular mechanisms of pulmonary fibrosis. Am J Physiol-Cell Physiol.

[3] Kandhare AD, Bodhankar SL, Mohan V (2015). Effect of glycosides based standardized fenugreek seed extract in bleomycin-induced pulmonary fibrosis in rats: decisive role of Bax, Nrf2, NF-kappa B, Muc5ac, TNF-alpha, and IL-1 beta. Chem Biol Interact.

[4] Dong XW, Jia YL, Ge LT (2017). Soluble epoxide hydrolase inhibitor AUDA decreases bleomycin-induced pulmonary toxicity in mice by inhibiting the p38/Smad3 pathways. Toxicology.

[5] Cui K, Kou JQ, Gu JH, et al. *Naja naja* atra venom ameliorates pulmonary fibrosis by inhibiting inflammatory response and oxidative stress. BMC Complement Altern Med. 2014;14(11). 10.1186/1472-6882-14-461. 10.1186/1472-6882-14-461 PMC4258260 25465226

[6] Tawfik MK, Makary S (2017). 5-HT7 receptor antagonism (SB-269970) attenuates bleomycin-induced pulmonary fibrosis in rats via downregulating oxidative burden and inflammatory cascades and ameliorating collagen deposition: comparison to terguride. Eur J Pharmacol.

[7] Behr J (2013). Evidence-based treatment strategies in idiopathic pulmonary fibrosis. Eur Respir Rev.

[8] Brown SW, Dobelle M, Padilla M (2019). Idiopathic pulmonary fibrosis and lung cancer a systematic review and meta-analysis. Ann Am Thoracic Society.

[9] Clevers H, Nusse R (2012). Wnt/beta-catenin signaling and disease. Cell..

[10] Niehrs C (2012). The complex world of WNT receptor signalling. Nat Rev Mol Cell Biol.

[11] Kim TH, Kim SH, Seo JY (2011). Blockade of the Wnt/beta-catenin pathway attenuates bleomycin-induced pulmonary fibrosis. Tohoku J Exp Med.

[12] Lam AP, Flozak AS, Russell S (2011). Nuclear beta-catenin is increased in systemic sclerosis pulmonary fibrosis and promotes lung fibroblast migration and proliferation. Am J Respir Cell Mol Biol.

[13] Hwang I, Seo EY, Ha H (2009). Wnt/beta-catenin signaling: a novel target for therapeutic intervention of fibrotic kidney disease. Arch Pharm Res.

[14] Sasaki M, Kashima M, Ito T (2000). Differential regulation of metalloproteinase production, proliferation and chemotaxis of human lung fibroblasts by PDGF, interleukin-1 beta and TNF-alpha. Mediat Inflamm.

[15] Kim C, Kim MC, Kim SM (2013). *Chrysanthemum indicum L*. extract induces apoptosis through suppression of constitutive STAT3 activation in human prostate cancer DU145 cells. Phytother Res.

[16] Li ZF, Wang ZD, Ji YY (2009). Induction of apoptosis and cell cycle arrest in human HCC MHCC97H cells with *Chrysanthemum indicum* extract. World J Gastroenterol.

[17] Kim IS, Ko HM, Koppula S (2011). Protective effect of *Chrysanthemum indicum Linne* against 1-methyl-4-phenylpridinium ion and lipopolysaccharide-induced cytotoxicity in cellular model of Parkinson's disease. Food Chem Toxicol.

[18] Aridogan BC, Baydar H, Kaya S (2002). Antimicrobial activity and chemical composition of some essential oils. Arch Pharm Res.

[19] Lee DY, Choi G, Yoon T (2009). Anti-inflammatory activity of *Chrysanthemum indicum* extract in acute and chronic cutaneous inflammation. J Ethnopharmacol.

[20] Cheng WM, Li J, You TP (2005). Anti-inflammatory and immunomodulatory activities of the extracts from the inflorescence of *Chrysanthemum indicum Linne*. J Ethnopharmacol.

[21] Wu XL, Li CW, Chen HM, et al. Anti-inflammatory effect of supercritical-carbon dioxide fluid extract from flowers and buds of *Chrysanthemum indicum Linnen*. Evid-based Complement Altern Med. 2013;13. 10.1155/2013/413237. 10.1155/2013/413237 PMC3816045 24223056

[22] Yang HM, Sun CY, Liang JL (2017). Supercritical-carbon dioxide fluid extract from *Chrysanthemum indicum* enhances anti-tumor effect and reduces toxicity of bleomycin in tumor-bearing mice. Int J Mol Sci.

[23] Zhang X, Xie YL, Yu XT (2015). Protective effect of super-critical carbon dioxide fluid extract from flowers and buds of *Chrysanthemum indicum Linnen* against ultraviolet-induced photo-aging in mice. Rejuvenation Res.

[24] Wu XL, Feng XX, Li CW, et al. The protective effects of the supercritical-carbon dioxide fluid extract of *Chrysanthemum indicum* against lipopolysaccharide-induced acute lung injury in mice via modulating toll-like teceptor 4 signaling pathway. Mediat Inflamm. 2014;13. 10.1155/2014/246407. 10.1155/2014/246407 PMC4158461 25214712

[25] Szapiel SV, Elson NA, Fulmer JD (1979). Bleomycin-induced interstitial pulmonary disease in the nude, athymic mouse. Am Rev Respir Dis.

[26] Liu Y, Wu H, Nie YC (2011). Naringin attenuates acute lung injury in LPS-treated mice by inhibiting NF-kappa B pathway. Int Immunopharmacol.

[27] Hosseini S, Imenshahidi M, Hosseinzadeh H (2018). Effects of plant extracts and bioactive compounds on attenuation of bleomycin-induced pulmonary fibrosis. Biomed Pharmacother.

[28] Shimbori C, Gauldie J, Kolb M (2013). Extracellular matrix microenvironment contributes actively to pulmonary fibrosis. Curr Opin Pulm Med.

[29] Lee JM, Dedhar S, Kalluri R (2006). The epithelial-mesenchymal transition: new insights in signaling, development, and disease. J Cell Biol.

[30] Jolly MK, Ward C, Eapen MS (2018). Epithelial-mesenchymal transition, a spectrum of states: role in lung development, homeostasis, and disease. Dev Dyn.

[31] Han Q, Lin LJ, Zhao BL (2018). Inhibition of mTOR ameliorates bleomycin-induced pulmonary fibrosis by regulating epithelial-mesenchymal transition. Biochem Biophys Res Commun.

[32] Su SD, Cong SG, Bi YK (2018). Paraquat promotes the epithelial-mesenchymal transition in alveolar epithelial cells through regulating the Wnt/beta-catenin signal pathway. Eur Rev Med Pharmacol Sci.

[33] Kolahian S, Fernandez IE, Eickelberg O (2016). Immune mechanisms in pulmonary fibrosis. Am J Respir Cell Mol Biol.

[34] Pan RY, Zhang YD, Zheng M, et al. Hydroxysafflor yellow a suppresses MRC-5 cell activation induced by TGF-beta 1 by blocking TGF-beta 1 binding to T beta RII. Front Pharmacol. 2017;8(12). 10.3389/fphar.2017.00264. 10.3389/fphar.2017.00264 PMC5425600 28553231

[35] Zhou XM, Wen GY, Zhao Y (2013). Inhibitory effects of alkaline extract of citrus reticulata on pulmonary fibrosis. J Ethnopharmacol.

[36] Song JS, Kang CM, Park CK (2013). Thrombin induces epithelial-mesenchymal transition via PAR-1, PKC, and ERK1/2 pathways in A549 cells. Exp Lung Res.

[37] Jiang F, Yang Y, Xue L (2017). 1 alpha,25-dihydroxyvitamin D3 attenuates TGF-beta-induced pro-fibrotic effects in human lung epithelial cells through inhibition of epithelial-mesenchymal transition. Nutrients..

[38] Robert S, Gicquel T, Victoni T, et al. Involvement of matrix metalloproteinases (MMPs) and inflammasome pathway in molecular mechanisms of fibrosis. Biosci Rep. 2016;36(11). 10.1042/bsr20160107. 10.1042/BSR20160107 PMC4945993 27247426

[39] Menou A, Duitman J, Crestani B (2018). The impaired proteases and anti-proteases balance in idiopathic pulmonary fibrosis. Matrix Biol.

[40] Yamashita CM, Radisky DC, Aschner Y (2014). The importance of matrix metalloproteinase-3 in respiratory disorders. Expert Rev Respir Med.

[41] Wang Y, Huang G, Mo B (2016). Artesunate modulates expression of matrix metalloproteinases and their inhibitors as well as collagen-IV to attenuate pulmonary fibrosis in rats. Genet Mol Res.

[42] Zhang HT, Fang SC, Wang CY (2015). MMP-9 1562C>T gene polymorphism and efficacy of glucocorticoid therapy in idiopathic pulmonary fibrosis patients. Genet Test Mol Biomark.

[43] Hamburg-Shields E, Dinuoscio GJ, Mullin NK (2015). Sustained beta-catenin activity in dermal fibroblasts promotes fibrosis by up-regulating expression of extracellular matrix protein-coding genes. J Pathol.

[44] Kiszalkiewicz J, Piotrowski WJ, Brzezianska-Lasota E (2017). Signaling pathways and their miRNA regulators involved in the etiopathology of idiopathic pulmonary fibrosis (IPF) and hypersensitivity pneumonitis (HP). Adv Respir Med.

[45] Liu F, Millar SE (2010). Wnt/beta-catenin signaling in oral tissue development and disease. J Dent Res.

[46] Clevers H (2006). Wnt/beta-catenin signaling in development and disease. Cell..

[47] Guo YT, Chen LW, Sun CY (2017). MicroRNA-500a promotes migration and invasion in hepatocellular carcinoma by activating the Wnt/b-catenin signaling pathway. Biomed Pharmacother.

[48] Yang SC, Liu Y, Li MY (2017). FOXP3 promotes tumor growth and metastasis by activating Wnt/beta-catenin signaling pathway and EMT in non-small cell lung cancer. Mol Cancer.

[49] Liu J, Ding X, Tang J (2011). Enhancement of canonical Wnt/beta-catenin signaling activity by HCV core protein promotes cell growth of hepatocellular carcinoma cells. PLoS One.

[50] Burgy O, Konigshoff M (2018). The WNT signaling pathways in wound healing and fibrosis. Matrix Biol.

[51] Baarsma HA, Konigshoff M (2017). 'WNT-er is coming': WNT signalling in chronic lung diseases. Thorax..

[52] Guo Y, Xiao L, Sun L (2012). Wnt/beta-catenin signaling: a promising new target for fibrosis diseases. Physiol Res.

[53] Willert K, Nusse R (1998). Beta-catenin: a key mediator of Wnt signaling. Curr Opin Genet Dev.

[54] Van Der Velden JLJ, Guala AS, Leggett SE (2012). Induction of a mesenchymal expression program in lung epithelial cells by wingless protein (Wnt)/beta-catenin requires the presence of c-Jun N-terminal Kinase-1 (JNK1). Am J Respir Cell Mol Biol.

[55] Pappas K, Xu J, Zairis S (2017). p53 maintains baseline expression of multiple tumor suppressor genes. Mol Cancer Res.

