# Supplementary material for: Effect of supercritical carbon dioxide fluid extract from Chrysanthemum indicum Linné on bleomycin-induced pulmonary fibrosis
Source: BMC Complement Med Ther. 2021 Sep 25;21:240. doi: 10.1186/s12906-021-03409-9 (PMC8464116; doi:10.1186/s12906-021-03409-9)
Supplement: Supplementary file 1 — Additional file 1. [file 12906_2021_3409_MOESM1_ESM.docx]

**Supplementary material 1**

**Effect of supercritical-carbon dioxide fluid extract from *Chrysanthemum indicum* Linné on Bleomycin-induced pulmonary fibrosis**

**Juan Nie^1, #^, Yanlu Liu^1, #^, Chaoyue Sun^2^, Jingna Zheng^1^, Baoyi Chen^1^, Jianyi Zhuo^1^, Ziren Su^1,3^, Xiaoping Lai^1,3^, Jiannan Chen^1, 3^, Jibiao Zheng^4, *^ and Yucui Li^1,3, *^**

**^1^****Mathematical Engineering Academy of Chinese Medicine, Guangzhou University of Chinese Medicine, Guangzhou 510006, China.**

**^2^ 2nd Clinical Hospital of Guangzhou University of Chinese Medicine, Guangzhou 510120, China.**

**^3^Guangdong Provincial Key Laboratory of New Drug Development and Research of Chinese Medicine, Guangzhou University of Chinese Medicine, Guangzhou 510006, China.**

**^4^ Department of Pharmacy, Central people’s Hospital of Zhanjiang, Zhanjiang 524000, China.**

**^#^ These authors contributed equally to this work**

**^*^** **These corresponding authors contributed equally to this work**

**Correspondence to: Jibiao Zheng,**

[**13828280428@163.com**](mailto:13828280428@163.com)

**Fax: 86 20 3935 8390**

**Yucui Li,**

[**liyucui@gzucm.edu.cn**](mailto:liyucui@gzucm.edu.cn)

**Fax: 86 20 3935 8390**

**The methods of GC-MS and HPLC-PAD analysis for the determination the compositions of CI_SCFE_**

This study aimed to determine the compositions of CI_SCFE_

**Materials and methods**

**Materials**

CI_SCFE_ was offered by the Institute of New Drug Research & Development Guangzhou University of Chinese Medicine (Lot. 20121104). GC-MS was offered by Agilent (Palo Alto, USA), HPLC was purchased from Hewlett Packard (Palo Alto, USA). Quercetin (Lot. 100081-200406), linarin (05-1017), apigenin (713-8712) and acacetin (480-44-4) were provided by Sigma-Aldrich Trading Co., Ltd. (Shanghai, China). Luteolin (130322) was purchased by Biotechnology Co., Ltd. (Chengdu, China), normal hexane, methyl alcohol, phosphoric acid, [acetonitrile](javascript:void(0);) and other chemicals were of analytical grade.

**Methods**

Taken the CI_SCFE_ 3.0 g, use the normal hexane (100 mL × 3) and 75% methyl alcohol (100 mL) ultrasound extraction three times and the layer of normal hexane were evaporated concentrated to 20 mL. The portion of normal hexane were analyzed by GC-MS and the other were analyzed by HPLC-PAD.

GC-MS was employed in the Agilent Technologies 7890B GC System, Chromatographic separation was achieved on a 5% phenyl methyl siloxane HP-5MS capillary column (30 cm × 250 μm × 0.25 μm, Agilent). The oven temperature was set initially at 60 ˚C followed by a gradient of 15 ˚C/min up to 180 ˚C (held for 6 min) and then programmed to 220 ˚C at 10 ˚C/min (held for 5min); furthermore, the temperature was up to 260 ˚C at 15 ˚C/min (held for 8 min) and finally to 280 ˚C at 10 ˚C/min (held for 5 min). No Split injecting samples (1 μL) and helium was used as carrier gas of 1.0 mL/min flow rate. The spectrometer was set in electron impact (EI) mode, the ionization energy was 70 eV, the scan range was 40-400 amu, and the scan rate was 0.34 s per scan. The inlet, ionization source temperatures were 230˚C and 250˚C, respectively. Identification of the compounds was based on a comparison of retention indices (relative to the retention times of n-alkanes on the HP-5MS column) and mass spectra with those of authentic samples, data from, the Wiley/NBS Registry of Mass Spectral Data (V.5.0), and the National Institute of Standards and Technology (NIST),and the MS Search (2015, V.2.0). The relative percentage of each compound in the normal hexane layer of CI_SCFE_ was quantified based on the peak area integrated by the analysis program.

HPLC-PAD analysis: HPLC analysis was employed in Agilent1100 HPLC system.

The separation was performed on a Kromasil KR100-5 C_18_ column (4.6 × 250 mm, 5 𝜇m, Kromasil) with a flow rate of 1.0 mL/min, column temperature at 30˚C, and injection volume of 10 𝜇L. The mobile phase consisted of acetonitrile (solvent A), and 0.1% Phosphoric acid (solvent B) was used to elute the targets with the gradient mode (0–5 min: 5% →25% A; 15–25 min: 25% →45% A; 25–28 min: 50% →60% A; 28–33 min: 60% →70% A; 33–40 min: 70% →5% A) . Analysis based on the retention time and the ultraviolet (UV) absorption (190 to 800 nm). The content of these compounds was quantitatively analyzed with peak areas under the standard curves at 334 nm.

**Results**

**GC-MS analysis of CI_SCFE_**

The results of GC-MS chromatograph of the normal hexane layers of CI_SCFE_ as shown in the **Fig. S1**, it clearly verified 30 compositions and all the components have shown in the **table S1**.

**HPLC analysis of CI_SCFE_**

The results of HPLC chromatograph of the 75% methyl alcohol layers of CI_SCFE_ as shown in the **Fig. S2**, it clearly verified 5 compositions have shown in the **table S2**.

**Table S1**: The chemical profile of CI_SCFE_, analyzed by GC-MS analysis and the relative percentage calculated by integrated peak area in Agilent MSD Chemstation data analysis system.

| **Number** | **Components** | **R.t** | **Percentage (%)** |
| --- | --- | --- | --- |
| **1** | **Camphor** | **5.570** | **0.559** |
| **2** | **L-. alpha. -Terpineol** | **6.033** | **1.565** |
| **3** | **9-Tetradecenal, (Z)** | **6.558** | **0.75** |
| **4** | **Thymol** | **6.861** | **0.505** |
| **5** | **n-Decanoic acid** | **7.594** | **1.181** |
| **6** | **Isoaromadendrene epoxide** | **9.843** | **0.784** |
| **7** | **Epiglobulol** | **10.858** | **1.011** |
| **8** | **Bornyltiglate** | **12.213** | **1.100** |
| **9** | **n-Propyl 5,8,11,14,17-eicosapentaenoate** | **12.917** | **0.993** |
| **10** | **Phytol** | **14.294** | **0.932** |
| **11** | **Hexadecanoic acid** | **17.236** | **0.813** |
| **12** | **Pentacosane** | **18.888** | **1.950** |
| **13** | **9,12-Octadecadienoic acid (Z, Z)** | **19.830** | **0.547** |
| **14** | **Oleic Acid** | **21.294** | **0.404** |
| **15** | **Tricosane** | **23.108** | **5.271** |
| **16** | **Tetracosane** | **24.831** | **0.803** |
| **17** | **Docosanoic acid** | **25.637** | **0.448** |
| **18** | **Tetratriacontane** | **26.189** | **6.341** |
| **19** | **α-Santonin** | **27.371** | **1.053** |
| **20** | **Pentacosane, 13-un20decyl** | **28.260** | **0.590** |
| **21** | **P21entacosane** | **29.027** | **8.62.3** |
| **22** | **Z-5-Methyl-6-heneicosen-11-one** | **30.426** | **0.572** |
| **23** | **Triacontane** | **30.663** | **1.337** |
| **24** | **Nonacosane** | **31.940** | **0.652** |
| **25** | **E, E, Z-1,3,12-Nonadecatriene-5,14-diol** | **33.212** | **13.491** |
| **26** | **Heptacosane** | **35.181** | **2.885** |
| **27** | **Dotriacontane** | **36.280** | **0.937** |
| **28** | **Octacosane** | **37.163** | **8.885** |
| **29** | **Octadecane, 3-ethyl-5-(2-ethylbutyl)** | **37.578** | **1.769** |
| **30** | **Hexatriacontane** | **39.055** | **1.602** |

**Table S2**: The chemical profile of CI_SCFE_, the relative percentage calculated by integrated peak area analyzed and quantified by HPLC-PAD analysis system.

| **Number** | **Components** | **R.t** | **Percentage (%)** |
| --- | --- | --- | --- |
| **1** | **Luteolin** | **16.918** | **0.725** |
| **2** | **Apigenin** | **17.313** | **0.883** |
| **3** | **Querocetin** | **21.135** | **0.85** |
| **4** | **Linarin** | **24.191** | **0.622** |
| **5** | **Acacetin** | **33.199** | **1.05** |


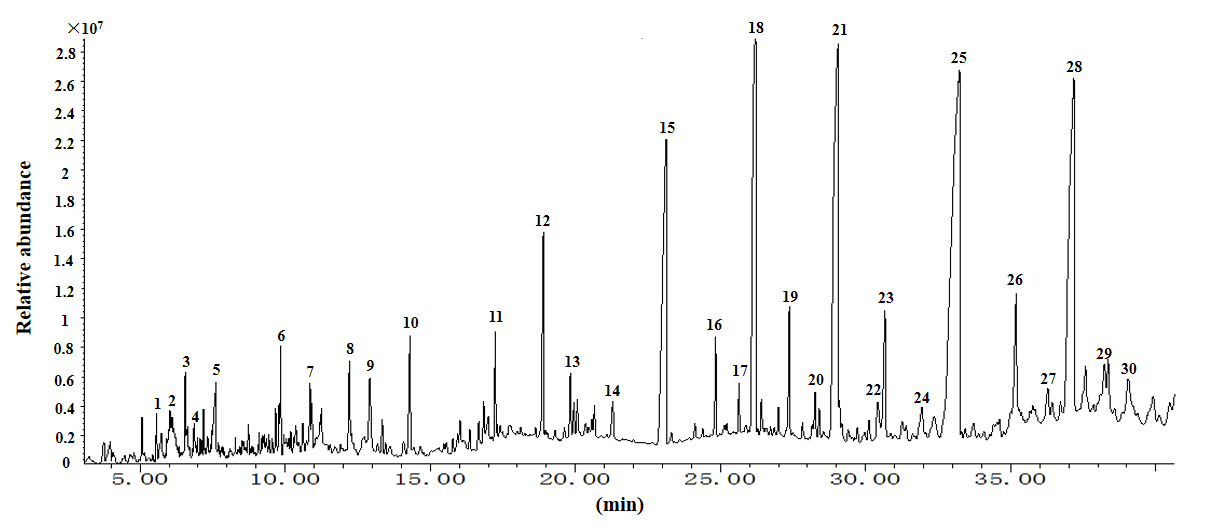


**Figure. S1**: The GC-MS chromatograph of the normal hexane layers of CI_SCFE_


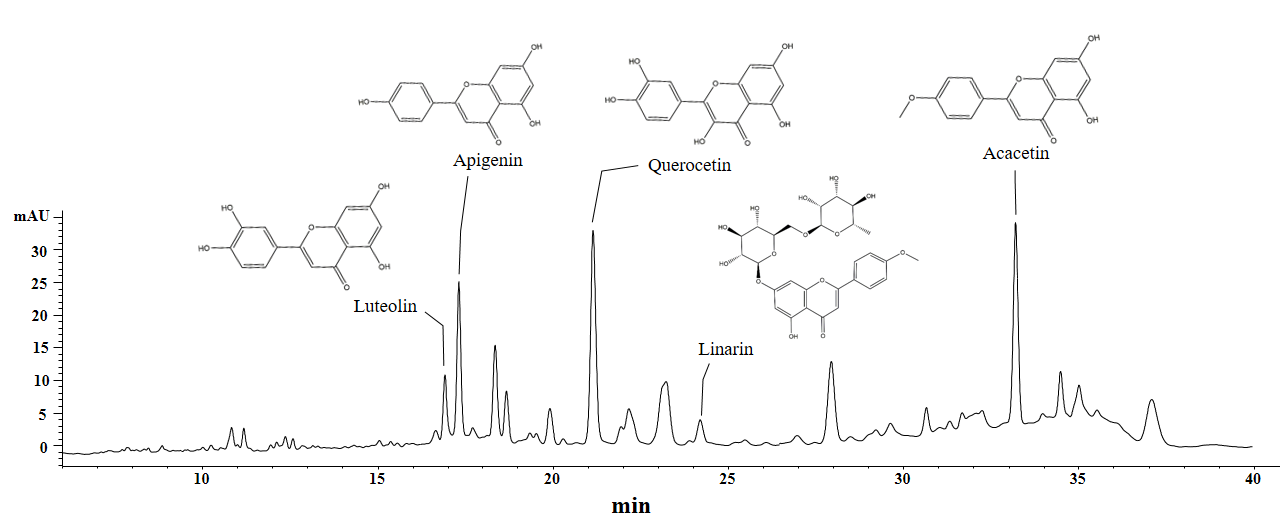


**Figure. S2**: The HPLC chromatograph of the 75% methyl alcohol layers of CI_SCFE_
