# Supplementary material for: Effect of supercritical carbon dioxide fluid extract from Chrysanthemum indicum Linné on bleomycin-induced pulmonary fibrosis
Source: BMC Complement Med Ther. 2021 Sep 25;21:240. doi: 10.1186/s12906-021-03409-9 (PMC8464116; doi:10.1186/s12906-021-03409-9)
Supplement: Supplementary file 2 — Additional file 2. [file 12906_2021_3409_MOESM2_ESM.docx]

**Supplementary material 2**

**Effect of supercritical-carbon dioxide fluid extract from *Chrysanthemum indicum* on BLM-induced pulmonary fibrosis**

**Juan Nie^1, #^, Yanlu Liu^1, #^, Chaoyue Sun^2^, Jingna Zheng^1^, Baoyi Chen^1^, Jianyi Zhuo^1^, Ziren Su^1,3^, Xiaoping Lai^1,3^, Jiannan Chen^1, 3^, Jibiao Zheng^4, *^ and Yucui Li^1,3, *^**

**^1^****Mathematical Engineering Academy of Chinese Medicine, Guangzhou University of Chinese Medicine, Guangzhou 510006, China.**

**^2^ 2nd Clinical Hospital of Guangzhou University of Chinese Medicine, Guangzhou 510120, China.**

**^3^Guangdong Provincial Key Laboratory of New Drug Development and Research of Chinese Medicine, Guangzhou University of Chinese Medicine, Guangzhou 510006, China.**

**^4^ Department of Pharmacy, Central people’s Hospital of Zhanjiang, Zhanjiang 524000, China.**

**^#^ These authors contributed equally to this work**

**^*^** **These corresponding authors contributed equally to this work**

**Correspondence to: Jibiao Zheng,**

[**13828280428@163.com**](mailto:13828280428@163.com)

**Fax: 86 20 3935 8390**

**Yucui Li,**

[**liyucui@gzucm.edu.cn**](mailto:liyucui@gzucm.edu.cn)

**Fax: 86 20 3935 8390**

**Preliminary experiment for** **the acute toxicity study**

Forty-eight Kunming (KM) mice (20-25g) were obtained from the Experimental Animal Center, Institute of Guangzhou University of Chinese Medicine. All animals were free to eat and drink. The procedures were approved by the Animal Care and Welfare Committee of Guangzhou University of Chinese Medicine. And the animal experiments were conducted according to the guidelines established by the National Institutes of Health (NIH) Guide for the Care and Use of Laboratory Animals.

KM mice were randomly divided into four group (12 mice per group, 6 male and 6 female) to evaluate the acute toxicity of the supercritical dioxide fluid extract from buds and flowers of *C. indicum* (CI_SCFE_) after a single oral dose. The mice were administered orally with CI_SCFE_ (0.5, 1, 2 and 4 g/kg). All experimental animals were fed with standard diet and clean water and kept under observation. The mortality or behavioral changes, including hyperactivity, tremors, ataxia, convulsions, salivation, diarrhea, lethargy, sleep, and coma, were regularly observed for 14 days.

After 14 days of a single oral dose, CI_SCFE_ did not cause any behavioral changes, and no mortality was observed. Therefore, the maximal tolerance dose (MTD) of CI_SCFE_ supposed to be larger than 4 g/kg in mice.

**Preliminary experiment for the** **effect of solvents and surgery on experimental rats**

Male adult Male adult Sprague-Dawley (SD) rats were obtained from the Experimental Animal Center, Institute of Guangzhou University of Chinese Medicine. All animals were free to eat and drink. The procedures were approved by the Animal Care and Welfare Committee of Guangzhou University of Chinese Medicine. And the animal experiments were conducted according to the guidelines established by the National Institutes of Health (NIH) Guide for the Care and Use of Laboratory Animals.

Thirty rats were divided into the control, sham operation control group, solvent control groups (10 rats per group). Rats in the control group, sham operation control group were treated with normal saline, rats in the solvent control groups were given normal saline (add 3% Tween 80 as a cosolvent). After 28 days of treatment, all rats in each group were weighed and delivered 10% chloral hydrate (0.5 mL/kg) with intraperitoneally anesthetized. Then, the rats were sacrificed, and the lungs were rapidly collected and washed with ice normal saline. Then 0.1g left lung tissues of all rats in each group were fixed with 4% formaldehyde. After fixed 24 h, lung tissues were embedded in paraffin and cut into 5 μm sections. Then sections were stained with hematoxylin and eosin (H&E) and Masson’s trichrome to observe the inflammation and collagen deposition.

As shown in Supp2. Figure S1, sham operation control group and solvent control groups exhibited no significant influence on the inflammation and collagen deposition when compared with control group.

**Preliminary experiment for the** **effect of CI_SCFE_ on A549, and MRC-5 cells**

A549 cells (1×104 cells/well) and MRC-5 cells (0.75×104 cells/well) were seeded in 96-well plates for MTS detection. Then, the medium was replaced with medium containing CI_SCFE_ (100, 200, 400, 800, 1000, 1600, 3200 ng/ml). MTS (20 μL) was employed after cells were cultured for 24/48 h. Four hours later, cell viabilities were measured at 492 nm using a multimode plate reader. The IC50 of CI_SCFE_ in the two cell lines was calculated using GraphPad Prism software (version 6).

As shown in Supp2. Figure S2, CI_SCFE_ significantly inhibited the proliferation of A549 cells (IC_50_: 2.723 ± 0.488 μg/mL) (**2A**) and MRC-5 cells (IC_50_: 2.236 ± 0.230 μg/mL) (**2B**).

**Preliminary experiment of** **Wnt-1 concentration selection**

A549 (1.25×10^5^ cells/mL) cells were seeded into 6-well plates. Then, the cells were stimulated with Wnt-1 (5, 10, 20, 40, 80 ng/mL), After incubation for 24 h, collected the cells and extracted the protein.

The proteins from the nuclear and cytoplasmic fractions of two cells and lung tissues were obtained with a Nuclear and Cytoplasmic Protein Extraction Kit (Keygen Biotech, Jiangsu, China). The protein concentrations of all the samples were estimated using the Bicinchoninic Acid Protein Kit (Best Bio, Shanghai, China). Fifty micrograms of two cells and lung tissues proteins were resolved using SDS-PAGE on 8% gels and were transferred onto PVDF membranes (Millipore, Billerica, USA). The membranes were blocked with 5% non-fat milk for 1 h. The membranes were incubated with anti-H3 (1:1500), anti-β-catenin (1:500) antibodies overnight at 4 ℃. Then, the membranes were treated with secondary antibodies (1:2000) for 2 h. Protein expression of two cells and lung tissues were measured by a chemiluminescence system (Tanon). The band densities of H3 were used as a reference.

As shown in Supp2. Figure S3, with the increase of Wnt-1 concentration, the expression of Nu-β-catenin protein in A549 cells gradually increased. Finally, 20 ng/ml Wnt-1 was selected for follow-up experimental research.

**Preliminary experiment of** **CI_SCFE_ concentration selection**

A549 (1.25×10^5^ cells/mL) cells were seeded into 6-well plates. Then, the cells were stimulated with Wnt-1 (20 ng/mL), when they reached 70%-80% confluence. After incubation for 24 h, cells were treated with medium containing CI_SCFE_ (100, 200, 400, 800 ng/mL). Then collected all the cells and extracted the protein after 24 h.

The proteins from the nuclear and cytoplasmic fractions of two cells and lung tissues were obtained with a Nuclear and Cytoplasmic Protein Extraction Kit (Keygen Biotech, Jiangsu, China). The protein concentrations of all the samples were estimated using the Bicinchoninic Acid Protein Kit (Best Bio, Shanghai, China). Fifty micrograms of two cells and lung tissues proteins were resolved using SDS-PAGE on 8% gels and were transferred onto PVDF membranes (Millipore, Billerica, USA). The membranes were blocked with 5% non-fat milk for 1 h. The membranes were incubated with anti-H3 (1:1500), anti-β-catenin (1:500) antibodies overnight at 4 ℃. Then, the membranes were treated with secondary antibodies (1:2000) for 2 h. Protein expression of two cells and lung tissues were measured by a chemiluminescence system (Tanon). The band densities of H3 were used as a reference.

As shown in Supp2. Figure S4, with the increase of CI_SCFE_ concentration, the expression of Nu-β-catenin protein in A549 cells gradually decreased. Finally, 400 ng/ml CI_SCFE_ was selected for follow-up experimental research.

**Figure Legends**


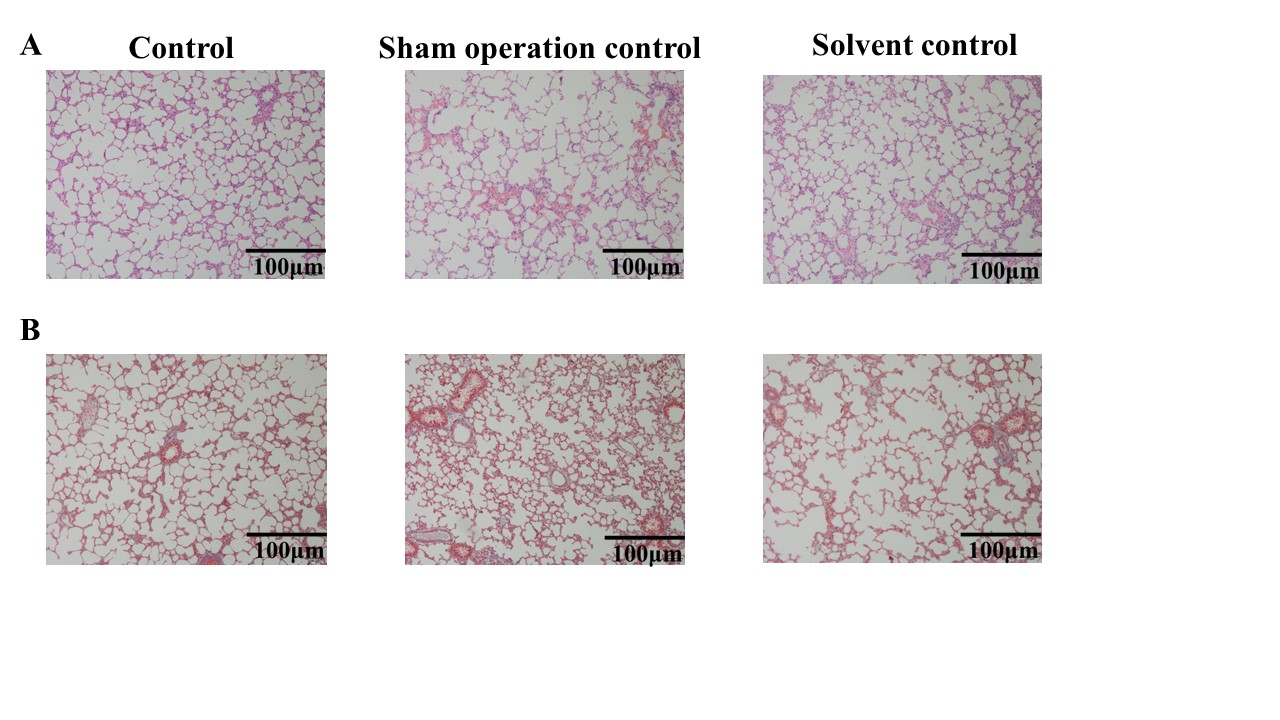
**Supp2. Figure S1. Rat Lung tissue changes.** Lung tissue sections were stained with hematoxylin-eosin (H&E) **(A)** for pathological examination; Masson **(B)** for collagen deposition; Scale bar indicates 100 μm.

**
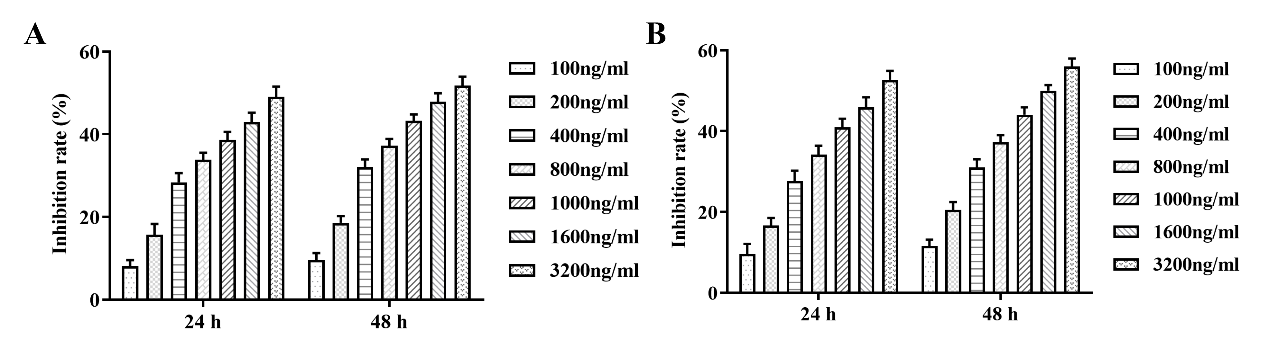
Supp2. Figure S2. Effect of CI_SCFE_ on A549, and MRC-5 cells.** A549 cells **(A)**, MRC-5 cells **(B)**

**Supp2. Figure S3. Effect of Wnt-1 on the expression of Nu-β-catenin in A549 cells.**

**^**^, *P* < 0.01 compared with control group.**

**
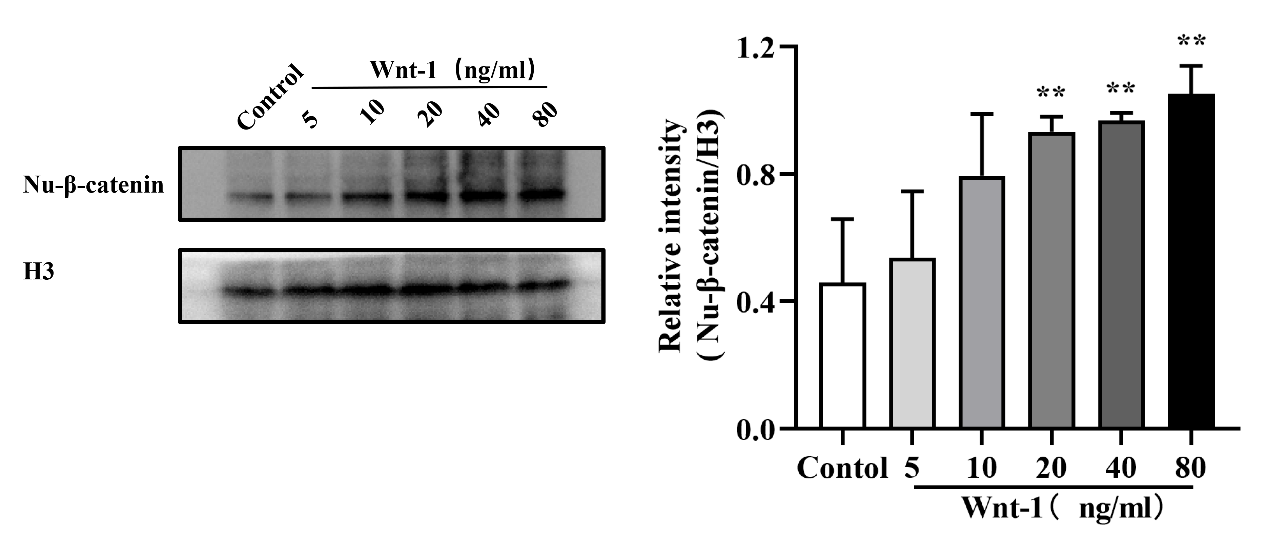
**

**Supp2. Figure S4. Effect of CI_SCFE_ on the expression of Nu-β-catenin in A549 cells .**

**^**^, *P* < 0.01; ^***^, *P* < 0.001 compared with Wnt-1 group.**

**
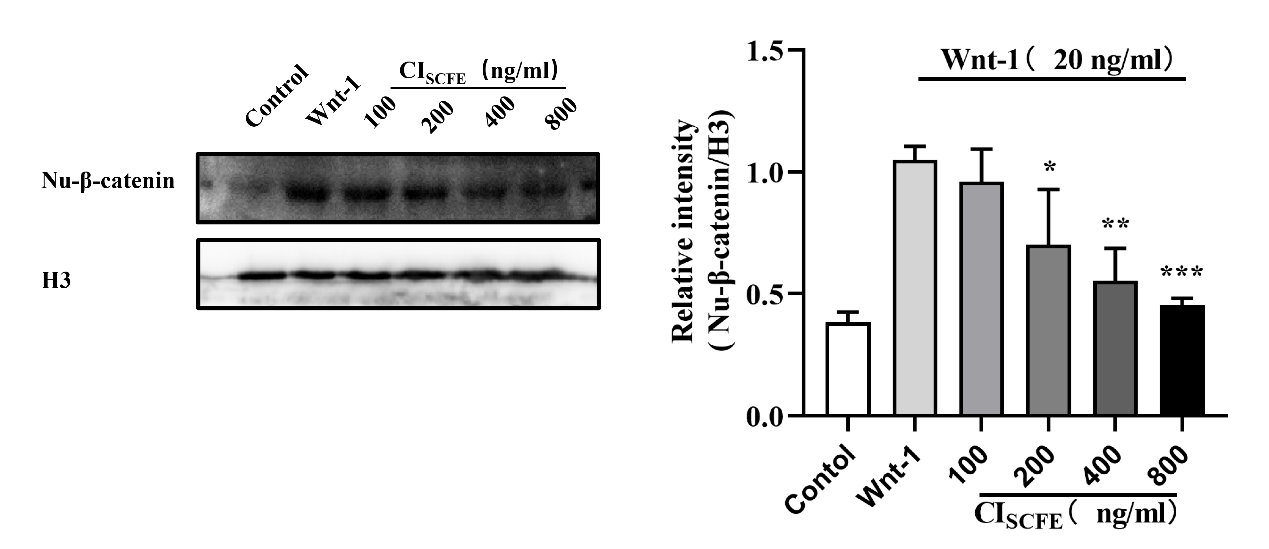
**
