# Supplementary material for: Effect of supercritical carbon dioxide fluid extract from Chrysanthemum indicum Linné on bleomycin-induced pulmonary fibrosis
Source: BMC Complement Med Ther. 2021 Sep 25;21:240. doi: 10.1186/s12906-021-03409-9 (PMC8464116; doi:10.1186/s12906-021-03409-9)
Supplement: Supplementary file 3 — Additional file 3. [file 12906_2021_3409_MOESM3_ESM.docx]

**Supplementary material 3**

**Effect of supercritical carbon dioxide fluid extract from *Chrysanthemum indicum* Linné on bleomycin-induced pulmonary fibrosis**

**Juan Nie^1, #^, Yanlu Liu^1, #^, Chaoyue Sun^2^, Jingna Zheng^1^, Baoyi Chen^1^, Jianyi Zhuo^1^, Ziren Su^1,3^, Xiaoping Lai^1,3^, Jiannan Chen^1, 3^, Jibiao Zheng^4, *^ and Yucui Li^1,3, *^**

**^1^****Mathematical Engineering Academy of Chinese Medicine, Guangzhou University of Chinese Medicine, Guangzhou 510006, China.**

**^2^ 2nd Clinical Hospital of Guangzhou University of Chinese Medicine, Guangzhou 510120, China.**

**^3^Guangdong Provincial Key Laboratory of New Drug Development and Research of Chinese Medicine, Guangzhou University of Chinese Medicine, Guangzhou 510006, China.**

**^4^ Department of Pharmacy, Central people’s Hospital of Zhanjiang, Zhanjiang 524000, China.**

**^#^ These authors contributed equally to this work**

**^*^** **These corresponding authors contributed equally to this work**

**Correspondence to: Jibiao Zheng,**

[**13828280428@163.com**](mailto:13828280428@163.com)

**Fax: 86 20 3935 8390**

**Yucui Li,**

[**liyucui@gzucm.edu.cn**](mailto:liyucui@gzucm.edu.cn)

**Fax: 86 20 3935 8390**

**A549 Cell**

**Note: The blots used in the manuscript figures were marked with black boxes.**

**Repeated blots were marked with red squares.**

**α-SMA:**

The expression of α-SMA in A549 cells (Figures 4 A)







The expression of α-SMA in transfected A549 cells (Figures 6 A)







**Collagen-I:**

The expression of Collagen-I in A549 cells (Figures 4 A)

**





**

The expression of Collagen-I in transfected A549 cells (Figures 6 A)

**

**





**Vimentin：**

The expression of Vimentin in A549 cells (Figures 4 A)

**



**

The expression of Vimentin in transfected A549 cells (Figures 6 A)

**



**

**Nu-β-catenin:**

The expression of Nu-β-catenin in A549 cells (Figures 4 A)

**





**

The expression of Nu-β-catenin in transfected A549 cells (Figures 6 A)

**



**

**H3:**

The expression of H3 in A549 cells (Figures 4 A)

**



**

The expression of H3 in transfected A549 cells (Figures 6 A)

**





**

**GAPDH:**

The expression of GAPDH in A549 cells (Figures 4 A)

**



**

The expression of GAPDH in transfected A549 cells (Figures 6 A)

**



**

**MRC-5 Cell**

**Note:**

**The blots used in the manuscript figures were marked with black boxes.**

**Repeated blots were marked with red squares.**

**α-SMA:**

The expression of α-SMA in MRC-5 cells (Figures 4 B)

**



**

The expression of α-SMA in transfected MRC-5 cells (Figures 6 B)

**



**

**Collagen-I:**

The expression of Collagen-I in MRC-5 cells (Figures 4 B)

**





**

The expression of Collagen-I in transfected MRC-5 cells (Figures 6 B)

**



**

**Vimentin：**

The expression of Vimentin in MRC-5 cells (Figures 4 B)

**



**

The expression of Vimentin in transfected MRC-5 cells (Figures 6 B)

**





**

**Nu-β-catenin:**

The expression of Nu-β-catenin in MRC-5 cells (Figures 4 B)

**





**

The expression of Nu-β-catenin in transfected MRC-5 cells (Figures 6 B)

**





**

**H3:**

The expression of H3 MRC-5 cells (Figures 4 B)

**



**

The expression of H3 in transfected MRC-5 cells (Figures 6 B)

**



**

**GAPDH:**

The expression of GAPDH in MRC-5 cells (Figures 4 B)

**



**

The expression of GAPDH in transfected MRC-5 cells (Figures 6 B)

**





**

**RAT**

**Note:**

**The blots used in the manuscript figures were marked with black boxes.**

**Repeated blots were marked with red squares.**

**α-SMA:**

The expression of α-SMA in rats (Figures 4 C)

**



**

**Collagen-I:**

The expression of Collagen-I in rats (Figures 4 C)

**





**

**Vimentin：**

The expression of vimentin in rats (Figures 4 C)

**





**

**Nu-β-catenin:**

The expression of Nu-β-catenin in rats (Figures 4 C)

**





**

**H3:**

The expression of H3 in rats (Figures 4 C)

**



**

**GAPDH:**

The expression of GAPDH in rats (Figures 4 C)

**



**
